# Multiple paternity and hybridization in two smooth-hound sharks

**DOI:** 10.1038/srep12919

**Published:** 2015-08-10

**Authors:** Ilaria A. M. Marino, Emilio Riginella, Michele Gristina, Maria B. Rasotto, Lorenzo Zane, Carlotta Mazzoldi

**Affiliations:** 1 Department of Biology, University of Padova, Via U. Bassi 58/B, 35121 Padova, Italy; 2 IAMC-CNR, via Luigi Vaccara 61, 91026 Mazara del Vallo (TP), Italy

## Abstract

Multiple paternity appears to be a common trait of elasmobranch mating systems, with its occurrence likely driven by convenience, due to females seeking to minimize the stress of male harassment. Here we use molecular markers to analyse the frequency of multiple paternity in two related viviparous sharks, *Mustelus mustelus* and *Mustelus punctulatus*. We first applied molecular methods to assign pregnant females, embryos and additional reference adults (N = 792) to one of the two species. Paternity analysis was performed using a total of 9 polymorphic microsatellites on 19 females and 204 embryos of *M. mustelus*, and on 13 females and 303 embryos of *M. punctulatus*. Multiple paternity occurs in both species, with 47% of *M. mustelus* and 54% of *M. punctulatus* litters sired by at least two fathers. Female fecundity is not influenced by multiple mating and in 56% of polyandrous litters paternity is skewed, with one male siring most of the pups. Genetic analyses also revealed hybridization between the two species, with a *M. punctulatus* female bearing pups sired by a *M. mustelus* male. The frequency of polyandrous litters in these species is consistent with aspects of their reproductive biology, such as synchronous ovulation and possible occurrence of breeding aggregations.

In the last 40 years, a substantial number of studies led to recognition that polyandry, i.e. females mating with different males within a breeding cycle, is a taxonomically widespread phenomenon, more frequent than expected in internal fertilizers^1^,^2^. The common occurrence of polyandry has raised theoretical and experimental attention to issues related to sexual selection and sexual conflict, such as the mechanisms involved in sperm competition and cryptic female choice as well as the costs and benefits that females may incur in multiple mating (for a review see^3^). Moreover, recent studies indicate that polyandry may also have consequences on population genetics, demography, and conservation^4^,^5^,^6^. Nonetheless, information on the frequency of polyandry in natural populations, crucial to deeply understanding its ecological and evolutionary role, is still limited^2^.

The reproductive biology of elasmobranchs, all internal fertilizers, is poorly known, mainly because the nature of their habitat makes it very difficult to observe mating in the field^7^,^8^. Only in the 21^st^ century has the use of molecular tools for parentage analysis provided evidence that female elasmobranchs mate with multiple males (reviewed in Fitzpatrick *et al.*^7^). Although limited to 22 out of the over 800 described species, genetic information on the elasmobranch mating system shows that multiple paternity is ubiquitous, occurring in all species (20 sharks and 2 skates) where more than one brood was analysed^7^,^9^,^10^,^11^,^12^. However, the frequency of polyandrous females, based on the number of multiply sired litters, appears to vary considerably both among and within species. Some species are predominantly polyandrous, with more than 80% of the broods sired by different males^13^,^14^,^15^,^16^, while in other species the majority (more than 80%) of broods are genetically monandrous^17^,^18^. Moreover, in the few cases in which different populations of a single species or the same population in different years have been analysed, the number of polyandrous females appears to vary geographically (*Carcharhinus plumbeus*^19^,^20^; *Squalus acanthias*^21^,^22^; *Mustelus henlei*^10^,^23^) and temporally (*Triakis semifasciata*^24^; *Mustelus henlei*^23^).

Frequency of genetic polyandry in elasmobranchs does not necessarily reflect the level of polyandrous mating behaviour^7^,^11^, since paternity may be highly influenced by post-copulatory and/or post-zygotic mechanisms^7^,^25^. Sperm storage, occurring in many elasmobranchs^26^, may facilitate post-copulatory influences on paternity by allowing either sperm competition, i.e. the competition among the ejaculates of different males, and/or cryptic female choice, i.e. the differential storage and utilization of sperm of different mates^7^,^27^. Alternatively, paternity may simply be the result of mating order, wherein the first male to mate with a female or the male that mates with a female closest to her ovulation fathers most or all of the pups. A direct indication that post-copulatory events affect the level of polyandry in elasmobranch comes from the observation that most litters show a skewed paternity, with one male achieving the greatest fertilization success^11^,^16^,^20^,^22^,^24^. However, the relative role of the different mechanisms potentially biasing paternity remains unclear.

The expected fitness advantage for females mating with multiple males^1^,^28^ has been evaluated in sharks by investigating direct benefits, in terms of increased fecundity^20^,^22^, as well as indirect genetic benefits, such as inbreeding avoidance^22^,^29^, offspring genetic diversity and survival rate^15^,^18^. However, no positive relationships have been found between any putative benefits for females and multiple paternity^11^. This has led to the hypothesis that shark polyandry is unavoidable, driven by the convenience for females to mate with multiple males in order to avoid or reduce harassment^15^. Female sharks incur strong mating costs, as they are often subjected to both external and internal injuries, with blood loss and increased risk of infection^30^. Thus, females would reduce the cost of male coercion by acquiescing to mating attempts^20^. According to the hypothesis of “convenience polyandry”, the major traits influencing polyandry frequency are female reproductive cycle and reproductive behaviour^11^,^18^. Female ovulation synchrony and behaviours that increase mate encounter rate, such as mating aggregations and female philopatry to breeding areas, which facilitate male exploitation of female receptivity, are expected to favour high polyandry frequency^11^.

Here we used 9 polymorphic microsatellite loci to assess the genetic mating system and the frequency of multiple paternity in 32 litters of two demersal Mediterranean smooth-hound sharks (Triakidae), whose reproductive biology is poorly known: the common smooth-hound, *Mustelus mustelus*, and the blackspotted smooth-hound, *Mustelus punctulatus*^31^.

Both species, whose morphological identification is problematic because most of the reported diagnostic characters partially overlap especially in early juvenile individuals^31^, inhabit the continental shelves and uppermost slopes, from the intertidal zone to more than 300 m of depth, on sandy, gravel and muddy substrates^31^,^32^. Females of both species store sperm for at least three months (Mazzoldi *et al.* unpublished), are placental viviparous and their reported fecundity ranges from 4 to 18 pups in *M. mustelus*, and from 12 to 27 pups in *M. punctulatus*^33^,^34^. They have a synchronized reproductive cycle with a gestation lasting 10–11 months and a period of 4–6 weeks between pregnancies, during which mating likely occurs^33^,^34^. In the Adriatic Sea, adults migrate predictably in space and time to give birth and then mate in coastal areas (Mazzoldi *et al.* unpublished). However, the spatial extent of the reproductive areas and their population density is unknown, but if the behaviour makes the location of receptive females more predictable, it may increase contact frequency with mating males.

The genetic mating system of these species has never been investigated; however, given the ubiquity of polyandry in all elasmobranchs studied so far, we expect that multiple paternity will occur. Given that these two sharks have a synchronized reproductive cycle, a short temporal gap between pregnancies, and mating areas in coastal zones where males and females might aggregate, we expect the frequency of polyandry to range from intermediate (around 50%) to high (over 80%). To evaluate the possible influence on paternity of post-copulatory events, we estimated the within-litter reproductive skew. Moreover, with the intent to highlight whether bias in paternity is more influenced by mating order or by sperm competition/cryptic female choice, we recorded the paternity of pups in relation to their position in the two uteri (right or left), within each uterus (postero-anterior order), and in relation to their size. If paternity is influenced by cryptic female choice, sperm competition, or simply mating order, the litters should be skewed to one male^35^. Since eggs are fertilized in sequence, the position of embryos along the postero-anterior axis of the uteri might reflect the order of fertilization^36^,^37^. In fact, if embryos do not move in the uterus, the older pups will occupy more posterior positions. In addition, in particular at early developmental stages, embryos generally display a size pattern along the axis of the uterus, with larger embryos located more posteriorly^36^,^37^. If skewed paternity is the result of mating order, we predict siblings to be spatially segregated in the uterus^38^ and, in the case of embryos movements within uterus, we still should observe similar sizes in siblings. Finally, to control for direct benefits associated with polyandry, i.e. increased fecundity, we analysed whether the number of pups, a proxy of female reproductive success, is influenced by the body size and/or the number of sires. As long as the mating system of these two smooth-hound sharks is consistent with that observed to date in elasmobranchs, we do not expect to find any positive relationship between polyandry level and female reproductive success.

## Results and Discussion

The application of two recently developed molecular methods^39^ allowed the unambiguous species identification as *Mustelus mustelus* or *M. punctulatus* of all the 32 pregnant females analysed and of 505 out of 507 embryos. Each specimen was characterized by the presence of a diagnostic amplification product of one of the two species obtained from the mitochondrial DNA (mtDNA) cytochrome c oxidase subunit 1 (COI)^39^, and by a corresponding species-specific multilocus genotype^39^ at 6 microsatellite loci (Table 1, Supplementary Table S1 and Supplementary Data S1). In particular (Table 1), two of these loci (Gg4 and MatJ5) were fixed between species, confirming previous results^39^. Three loci (Gg20, Mca33 and Mh1) had low variation and non-overlapping allele distribution; respect to previous findings^39^, locus Mh1 showed in this larger dataset a new rare allele restricted to *M. punctulatus*. The McaB26 locus, previously reported to have partial allelic overlap but to discriminate the two species based on species-specific genotypes^39^, was less informative in this study due to the finding of genotypic overlap in one individual.

Accordingly, 19 females were classified as *M. mustelus* (TL: 1210–1630 mm; litter size range: 3–18 embryos) and 13 as *M. punctulatus* (TL: 1100–1410 mm; litter size range: 9–35 embryos) (Table 2 and Supplementary Data S1). In addition, 204 embryos were classified as *M. mustelus* and 301 as *M. punctulatus*. In all these cases, as expected, the results of the embryos’ molecular identification were fully consistent with those obtained for the corresponding mother (see Supplementary Data S1).

However, two out of 507 embryos, collected from one single *M. punctulatus* female (Mp1_6.9) sampled in the northern Adriatic Sea, besides being classified as *M. punctulatus* using the mtDNA COI test, showed a mixed genotype at the 6 microsatellite loci used for identification^39^ (Table 1 and Supplementary Data S1). This pattern was particularly evident at loci Gg4 and MaTJ5 where the two pups showed an heterozygote genotype, with an allele typical of *M. mustelus* and the other of *M. punctulatus*, despite the loci are fixed for different alleles within each species as reported in Marino *et al.*^39^ and further confirmed in this study (Table 1 and Supplementary Data S2). Similarly, for loci Gg20, Mca33 and Mh1, slightly polymorphic within species with no allelic overlap between species (Table 1 and Supplementary Data S2), the genotype of these two individuals was heterozygote with one *M. mustelus* and one *M. punctulatus* allele. Locus McaB26 was not informative for hybrid identification because of allelic and genotypic overlap between the two species (Table 1 and Supplementary Data S2). Given the existence of alleles differing for one single base in two of the 6 loci used for identification (Gg4 and Gg20, Table 1), which is likely due to indels in the flanking regions as a result of cross-amplification from relatively distant species (Supplementary Table S1), we confirmed the hybrid nature of these two pups by the amplification of a region of the ribosomal Internal Transcribed Spacer 2 (ITS2), containing a species diagnostic length polymorphism as reported in Marino *et al.*^39^. ITS2 amplification produced the two-band phenotype expected in hybrids for the two embryos under investigation, whereas their siblings showed the one band phenotype typical of *M. punctulatus*^39^ (Supplementary Fig. S1). Therefore, we believe that the two embryos can be confidently classified as hybrids.

A set of 253 additional adult samples was also subjected to genetic identification, allowing the unambiguous classification of 136 *M. mustelus* and 117 *M. punctulatus* (Table 1, Supplementary Data S2). These samples were used to provide estimates of the genetic diversity of the two species and assess the power to detect multiple paternity using a different set of 9 microsatellites, previously shown to be polymorphic within species^39^. Summary statistics for polymorphic loci are reported in Table 3. As expected from Marino *et al.*^39^, 5 loci (MaD2X, McaB5, McaB35, Mh9 and Mh25) were successfully amplified in both species. Two further loci (Gg22 and MaFYP) amplified only in *M. mustelus*, and two (MaND5 and Mh29) only in *M. punctulatus*, leading to a total of 7 useful loci for each species. In *M. mustelus*, the number of alleles per locus ranged from a minimum of 3 (Mh9) to a maximum of 13 (McaB35), whereas the observed and expected heterozygosity ranged from 0.184 (Mh25) to 0.852 (McaB35) and from 0.218 (Mh25) to 0.831 (McaB35) respectively. In *M. punctulatus*, alleles ranged from a minimum of two (McaB5) to a maximum of 5 (MaND5) per locus, with the observed and expected heterozygosity varying from 0.017 (McaB5) to 0.709 (MaND5) and from 0.017 (McaB5) to 0.686 (MaND5) respectively. All microsatellite loci conformed to the expectations of HWE (all P-values > 0.01 after Bonferroni correction) and tests for linkage disequilibrium among all loci pair did not show significant departures from expected values.

Polymorphic loci confirmed the molecular species identification reported above and provided further support for the existence of two hybrid embryos. In fact, a Bayesian clustering analysis, based on the 4 loci that successfully amplify and show the highest polymorphism in both species (MaD2X, McaB35, Mh9 and Mh25, Table 3), clearly indicated the presence of two non-mixing groups of genotypes, corresponding to the two species, and identified admixture in the two hybrid embryos only (Supplementary Fig. S2).

The power to detect multiple paternity was estimated with the PrDM software^40^ taking into account the allelic frequencies observed at the 9 polymorphic loci (7 for each species) in the additional adults and the litter size of each pregnant female. This analysis indicated that the probability of detecting multiple matings was generally high and rapidly increased with both litter size and the number of sires in the two species (Table 4). In *M. mustelus*, the power of accurately assessing the number of fathers was relatively low only for the smallest litter collected (3 embryos), ranging from 40 to 60%, but increased to 95–100% with moderate or large litter size (9–18 embryos). In *M. punctulatus*, a litter of as few as 9 embryos (the minimum litter size sampled) was sufficient to get a power ranging from 71% to 90%, with the power reaching 98% when considering a brood size of 35.

Results of the paternity analysis are reported in Table 5. Nine out of 19 litters (47%) of *M. mustelus* and 7 out of 13 (54%) of *M. punctulatus*, were sired by multiple males and a maximum of three sires per litter was detected by allele count with the program GERUD. The remaining litters (10 of *M. mustelus* and 6 of *M. punctulatus*) were monandrous, with sires contributing a maximum of two alleles per locus per brood; in these litters there was no evidence for departure from the expected Mendelian segregation of paternal alleles since Fisher’s method combined multilocus probability were always above 0.05 (16 litters; *χ*^2^ range: 0.288–16.122; d.f. = 14; P-values range 0.305–1.000). Considering the specific PrDM values calculated for each monandrous litter, it is unlikely that multiple paternity has been undetected in these broods, except for the three smallest families of *M. mustelus* (Table 2, Supplementary Table S2).

The expected Bayesian frequency of multiple mating, estimated with the software FMM^41^, was 45% (confidence interval CI: 14%–76%) in *M. mustelus* and 53% (CI: 27%–80%) in *M. punctulatus*, closely matching the values of 47% and 54% obtained from direct count of non-maternal alleles.

The frequency of polyandrous litters in our study species is in line with the values reported for other triakid sharks, varying from 31 to 58%^11^,^24^,^38^. Considering the low sample size per site and year and the wide confidence intervals of our estimates, geographical and temporal variations in paternity in the two species were not analysed.

In polyandrous litters, the average number of sires was 2.1 for *M. mustelus* and 2.0 for *M. punctulatus*. Considering that females of the genus *Mustelus* can store sperm in specialized organs, the oviducal glands^42^,^43^,^44^,^45^, and sperm longevity is unknown, the observed genetic polyandry would not necessarily reflect the occurrence of female polyandrous behaviour within a breeding season. Indeed, bi-sired litters could be the result of a delayed fertilization of sperm accumulated in the previous breeding season rather than of female mating with multiple males during the course of a single reproductive event. However, since the behavioural observations of shark mating in the field always reported female multiple mating and male harassment^30^,^46^,^47^, the multiple paternity recorded in this study is likely to result from female polyandrous behaviour.

In a possible scenario of female polyandrous behaviour, the common occurrence of two fathers per litter may reflect a limited number of copulations, and/or the influence of post-copulatory mechanisms on paternity. As stated above, we cannot speculate on the mate encounter rate and the level of female polyandrous behaviour. However, our results suggest that post-copulatory mechanisms influence the within-litter number of fathers. In species where females store sperm, a predicted outcome of post-copulatory mechanisms, such as sperm competition, cryptic female choice or simply mating order, is the occurrence of a skewed paternity^35^. Consistent with this hypothesis, in both of the species studied, the within-litter paternity was significantly skewed in 5 of the 9 litters of *M. mustelus* and 4 of the 7 litters of *M. punctulatus*, with one male siring on average 69 ± 19% and 70 ± 17% of the embryos respectively.

However, male reproductive success was not mirrored by embryos position and size. In fact, in *M. mustelus*, the species where it was possible to analyse spatial distribution of embryos, the paternity pattern did not differ within the uterus neither along the postero-anterior axis (Wald χ^2^ = 18.658, d.f. = 16, P = 0.287) nor considering the embryo size rank (Wald χ^2^ = 24.094, d.f. = 16, P = 0.087). With this regard, it is worth noticing that the analysed embryos were collected from August to early October, and were therefore in the first half of their development^33^ (see also embryo size in Supplementary Data S1); thus, most of the embryos were still presenting a well-developed yolk sac, and were then in a developmental stage in which differences in size among embryos are particularly pronounced and might be linked to insemination order^37^. If embryos position and size reflect the order of insemination, our non-significant results would suggest that male reproductive success is not primarily driven by sperm precedence. This hypothesis, however, should be considered with caution and requires further support, since our results have been obtained from few litters only (Supplementary Data S1). Moreover, in addition to fertilization order, other factors may affect embryo position within uteri (for instance possible movements of embryos within uteri) or embryo size rank (for instance food supply to each embryo^36^).

Our results also showed that paternity did not differ between uteri (Wald χ^2^ = 0.167, d.f. = 1, P = 0.683). In elasmobranchs, males insert one clasper per copulation, and Pratt and Carrier^48^ hypothesized that the delivery of sperm to each oviduct is independent. This would imply that, in the studied species, each sire would have performed successive copulations. A more parsimonious explanation for the presence of siblings in both uteri relies on the anatomy of female genitalia. Indeed, as left and right oviducts open in the common chamber of the cloaca, it is likely that the propulsion of sperm into the cloaca, favoured by the siphon sac^49^, would allow the simultaneous delivery of sperm to the two oviducts and the respective oviducal glands.

Multiple mating does not influence fecundity, as in both species litter size was related to female size but not to the occurrence of polyandry (*M. mustelus*, ANCOVA: female size: F_2,16_ = 12.265, P = 0.003; mating system: F_2,11_ = 0.503, P = 0.488; *M. punctulatus*, ANCOVA: female size: F_2,10_ = 5.306, P = 0.044; mating system: F_2,10_ = 0.138, P = 0.718). This result agrees with the findings of previous studies^11^,^15^,^18^,^20^,^22^,^29^ and it supports the idea that multiple mating in elasmobranchs is not shaped by direct benefits in terms of fertility.

The alternative hypothesis of “convenience polyandry”, under which females are acquiescent to matings to reduce the cost of coercion by males, seems more likely, considering also the identification in this study of a case in which a female mated with an individual of a different species. In fact, as mentioned before, two pups of one *M. punctulatus* female (Mp1_6.9), sampled in the northern Adriatic Sea, were confidently classified as hybrids. To our knowledge, this is the second study in which hybridization is recorded in the class Chondrichthyes; the first involving two black tip sharks *Carcharhinus tilstoni* and *C. limbatus*, in an area where they overlap along the eastern Australian coastline^50^. In black tip sharks, hybrids were detected both among juveniles and adults, and it has been suggested that they may be fertile and even have some ecological advantages over one of the two species, at least in some parts of their spatial distribution^50^. Our study species also have a highly overlapping distribution, but we found only two hybrids out of 507 embryos and no evidence of hybridization in adults, which may suggest reduced hybrid viability. Therefore, future studies with large-scale genetic datasets and reproductive life-history characters are needed to better understand the extent and direction of the hybridization between the two species.

## Conclusions

We detected multiple paternity in both *Mustelus mustelus* and *M. punctulatus*, confirming our expectations based on the ubiquitous presence of polyandrous litters in elasmobranch species^7^,^9^,^10^,^11^,^12^. The presence of pups sired by different males suggests that females may mate with multiple males over the course of a single breeding cycle. In both study species, around half of the litters are sired by multiple males, a frequency intermediate to those reported in other elasmobranchs where the great majority of the litters are either monandrous^17^,^18^ or polyandrous^13^,^15^,^16^,^20^. In this taxon, female polyandry varies both among species and between populations^19^,^20^,^23^, with different factors, ranging from reproductive cycle to demographic processes and post-copulatory sexual selection, potentially influencing its frequency^7^,^11^,^14^. An ovulation period protracted for several months has been suggested to favour multiple paternity by increasing the opportunity of successive inseminations over an extended period of time^14^. In fact, oviparous species where the ovulation-fertilization cycle may last for several months show high rates of multiple paternity^14^,^16^. Nonetheless, similar levels of polyandry have been recorded also in viviparous species, which usually have a short ovulation window^10^,^13^,^14^,^15^,^20^. In elasmobranchs, polyandrous behaviour seems to be the result of convenience, with females capitulating to multiple matings simply to avoid the physical costs associated with resisting or avoiding copulations^15^,^20^. In this study, we did not find evidence that multiple paternity increased fecundity. Instead, some of the reproductive modalities of *Mustelus* species, such as female ovulation synchrony, a short period between successive pregnancies, and the occurrence of breeding areas where males may easily predict female receptivity, concur to suggest that in the study species as well, females may engage in convenience polyandry. Moreover, the presence of hybrid pups in a female of *M. punctulatus* could be a further indication that males harass females, irrespective of the potential mate species, driving them to submit to copulations.

Under the scenario of convenience polyandry, the frequency of multiple paternity may depend on population demography, influencing the number of mate encounters^51^ and/or post-copulatory mechanisms^7^,^11^. In our study species, lacking information on the population demography in breeding sites, we cannot evaluate the possible weight of mate encounter rate on the frequency of multiple paternity. However, in half of the polyandrous litters, most of the pups were sired by a single male, an expected outcome if post-copulatory mechanisms influence paternity success^35^. Future combined investigation on the reproductive modalities, demography, morphology of sperm storage organs, and sperm morphology and performance are needed to decipher the relative influence of reproductive behaviour (i.e. relative abundances of males and females in breeding areas) and post-copulatory sexual selection in shaping the frequency of polyandry among shark species/populations.

## Methods

### Sampling and DNA extraction

Mother-embryo samples (32 pregnant females and 507 embryos) were collected in the northern Adriatic Sea (offshore Chioggia and Ancona) and in the Strait of Sicily (close to Mazara del Vallo) between 2008 and 2013, from local fishermen, scientific surveys on board commercial vessels, and scientific surveys of the projects MEDITS (International Bottom Trawl Survey in the Mediterranean) and Campbiol (Table 2). No experiments on living animals were performed; all the analyses were carried out on dead specimens caught during fishing activity and, therefore, no approval from the local ethics committee was necessary. The total length (TL) of each female was measured to the nearest mm; moreover, in a subsample of the mother-embryo samples collected in 2012 and 2013, we recorded the size and the location of each embryo in the two uteri (left or right) and its position along the postero-anterior axis of the uteri. In order to record the position, the uteri were carefully removed from the female and ventrally opened. Embryos were horizontal or diagonally oriented within the uterus, allowing a clear determination of their order along the postero-anterior axis. Embryos were individually removed and sampled from each uterus and their size measured (to the nearest mm, with a digital caliper). A rank of order (from posterior to anterior) and a rank of size (from larger to smaller) were then attributed to each embryo for the following statistical analyses. Embryos showed a size pattern along the postero-anterior axis, with the largest ones at the posterior end of the uteri (see Supplementary Data S1).

Additional adult samples from the same sites (N = 253) were included in the analysis, to provide estimates of the genetic diversity of the two species and to test the power to detect multiple paternity.

A fin clip or a muscle sample was collected from each specimen and preserved in 95% ethanol. Genomic DNA was extracted using a standard salting out protocol^52^ and stored at −20 °C before PCR amplification.

### Molecular species identification

Before starting multiple paternity analyses, all the specimens (pregnant females, embryos and additional adults, total N = 792) were genetically identified as *M. mustelus* or *M. punctulatus*, using two previously developed molecular methods^39^. Briefly, we used: 1) a mitochondrial DNA assay based on species-specific amplification of a fragment of the cytochrome c oxidase subunit 1 (COI) using primers matching diagnostic sequence polymorphisms; and 2) a microsatellite method based on 6 loci, originally isolated from other sharks^53^,^54^,^55^,^56^, which have been shown to have a limited polymorphism or to be fixed within *M. mustelus* and *M. punctulatus*, and to bear a different genotypic distribution in the two species (see Marino *et al.*^39^ for experimental conditions and Supplementary Data S1 and S2 for genetic information). Importantly, the microsatellite method, being based on nuclear DNA, allows the quick identification of hybrids, if any.

### Genetic assessment of multiple paternity

To detect multiple paternity, we used 9 microsatellite loci, originally isolated from other shark species^10^,^53^,^54^,^55^,^56^ (Supplementary Table S1), which were shown to cross-amplify and to be polymorphic in *M. mustelus* and/or *M. punctulatus*^39^; the final panel, considering that two loci work in *M. mustelus* only and two in *M. punctulatus* only, consisted of 7 polymorphic loci per species.

Forward primers of all 9 loci were labelled with fluorescent dyes for detection with ABI PRISM automatic sequencer and amplified together in a single multiplex reaction. PCRs were carried out in 10 μL total volume containing: 1X QIAGEN Multiple PCR Master mix (QIAGEN, HotStarTaq DNA Polymerase, Multiplex PCR Buffer, dNTP Mix), 0.2 μM primer mix and 100 ng of DNA template. PCR conditions were as follows: initial activation step for 15 min at 95 °C, 30 cycles of 30 s at 94 °C, 90 s at 57 °C and 1 min at 72 °C, and final elongation for 30 min at 60 °C. PCR products were run on an ABI 3100 automated sequencer and sized with the standard LIZ500 (Applied Biosystems).

Fragment analysis was performed at the BMR Genomics Molecular Biology service (www.bmr-genomics.it) and microsatellite analysis was carried out using PEAK SCANNER ver. 1.0 (Applied Biosystems). Ten percent of all specimens were re-genotyped and rescored manually to detect potential scoring errors. No discrepancies were found. In order to minimize the negative consequences of poor allele calling, we automated binning with the software FLEXIBIN ver. 2^57^ and manually checked the final scoring to ensure the accuracy of the process (see Supplementary Data S1 and S2 for microsatellite genotypes).

### Statistical analyses

For the 9 polymorphic loci used for multiple paternity (Supplementary Table S1), the number of alleles (N_a_), the observed (H_o_) and the unbiased expected heterozygosity (H_e_) were estimated, using GENETIX ver. 4.05.2^58^, on the 253 adults after their molecular species identification. We also tested for deviation from Hardy-Weinberg equilibrium (HWE) at each locus, and for linkage disequilibrium between pairs of loci using GENEPOP ver. online 4.0.10^59^,^60^. Significance of all tests was estimated by the Markov Chain method (dememorizations = 10000, batches = 500, iterations = 10000).

In order to confirm the molecular species identification and to provide further support for the existence of hybrids, a Bayesian clustering analysis was performed. To this end, we used the 4 of the 9 polymorphic loci that successfully amplify in both species and show the highest polymorphism (MaD2X, McaB35, Mh9 and Mh25, Table 3). The program STRUCTURE 2.3.4^61^,^62^,^63^ was used to estimate the most likely number of genetic clusters, ignoring a priori information about species assignment of individuals, and to estimate individual admixture proportions. The analysis was performed on 270 individuals (family Mp1_6.9 with 16 pups, 136 adults of *M. mustelus* and 117 adults of *M. punctulatus*). STRUCTURE was run with 10 independent simulations for each value of K from 1 to 3, each with 1000000 iterations, following a burn-in period of 100000 iterations. In all simulations, individual admixture proportions and their 95% posterior probability intervals were calculated by assuming an admixture ancestry model (i.e. allowing the genetic composition of individuals to be a mixture from different groups) and with uncorrelated allele frequencies.

The probability of detecting and correctly quantifying multiple paternity within brood was calculated with the PrDM program^40^. We ran a number of simulation scenarios to test the power afforded by our suite of microsatellite loci to unveil multiple paternity in *M. mustelus* and *M. punctulatus* under four different hypothetical scenarios: (1) two males with equal breeding success, (2) two males with skewed success (66.7% and 33.3%), (3) three males with equal breeding success and (4) three males with skewed success (57%, 28.5% and 14.5%). As the probability of detecting multiple mating is also a function of the number of offspring analysed, we ran PrDM simulations with litter sizes ranging from 3 to 18 (the minimum and maximum clutch sizes observed in the present study for *M. mustelus*) and from 9 to 35 (the minimum and maximum observed in our samples for *M. punctulatus*).

Analysis of paternity was carried out with the GERUD 2.0 software^64^ to estimate the minimum number of sires through an exhaustive search. The known maternal genetic contribution was subtracted from the genotype of the embryos at each locus and the number of paternal derived alleles was then summed. A litter that had three or more paternal alleles at one or more loci was considered polyandrous. When a unique sire-progeny solution was not obtained, we ranked the alternative solutions according to the relative probability^64^. For each monandrous litter, further evidence for the existence of a single sire was obtained by testing Mendelian inheritance of paternal alleles. To this end, we performed for each locus/litter a *χ*^2^ - goodness of fit test against an expected 1:1 inheritance ratio; single locus results were combined for each litter using Fisher’s method^65^.

The Bayesian program FMM^41^ was used to estimate expected frequency of multiple paternity in the two species. This method uses a Bayesian model that incorporates the number of loci in the analysis, their allele frequencies, the maternal genotype, the number of paternal alleles in each clutch and the prior probability of multiple mating. Taking into account this information, it calculates the most likely frequency of multiple mating, and assigns a 95% confidence interval to that estimate^41^.

We tested the effect of mating system (monandrous vs polygynous) on litter size with ANCOVA analysis of covariance, with mating system as a fixed factor with two levels (monandry and polyandry), and female size as a covariate.

We tested the spatial segregation of siblings between uteri by applying a generalized linear model with the father identity nested in the mother as predictor variable, and uterus as response variables using a binary logistic fit.

The spatial segregation was further investigated within uteri, using generalized linear models, with the intent to provide indications on the occurrence of fertilization order on siblings. This analysis was performed on the litters of *M. mustelus*, collected in 2012 and 2013, for which within uterus embryo information was available and multiple paternity was detected (Supplementary Data S1). We used two measures as response variables, namely the embryo position within uteri and the embryo size rank, this latter to take into account the possibility that embryos might move within uterus; as before, the father identity nested in the mother was the predictor variable, and an ordinal logistic fit was used.

## Additional Information

**How to cite this article**: Marino, I.A.M. *et al.* Multiple paternity and hybridization in two smooth-hound sharks. *Sci. Rep.* **5**, 12919; doi: 10.1038/srep12919 (2015).

## Supplementary Material

Supplementary Information

Supplementary Dataset 1

Supplementary Dataset 2

## Figures and Tables

**Table 1:** Microsatellite loci for *Mustelus mustelus*and *M. punctulatus*for molecular identification.

Locus	Repeat size	Mm	Mp	Hybrids
Gg4	1	198/198	199/199	198/199
Gg20	1	280/280	281/281281/282282/282	280/282280/281
MaTJ5	2	159/159	157/157	157/159
Mca33	3	194/194194/200200/200	197/197	197/200
McaB26	5	224/224224/229229/229	224/224	224/224224/229
Mh1	2	203/203	201/201201/205	201/203

Reported are: locus name, repeat size determined from raw size data by Flexibin^57^, and genotypes observed in *M. mustelus* (N = 359), *M. punctulatus* (N = 431), and in the two hybrid embryos referred in the main text. Mm = *M. mustelus*; Mp = *M. punctulatus*.

**Table 2:** Female total length (TL), litter size (No. embryos), sampling locations and date of capture for the 32 litters.

Species	Mother	TL (mm)	No. embryos	Sampling location	Sampling date
*M. mustelus*	Mm152	1630	10	Mazara del Vallo	12.2013
	Mm155	1270	4	Mazara del Vallo	12.2013
	Mm156	1410	4	Mazara del Vallo	12.2013
	Mm159	1350	6	Mazara del Vallo	12.2013
	Mm161	1210	3	Mazara del Vallo	12.2013
	Mm203	1550	16	Chioggia	11.09.2012
	Mm229	1585	18	Mazara del Vallo	17.09.2012
	Mm230	1490	15	Mazara del Vallo	17.09.2012
	Mm235	1420	7	Chioggia	26.09.2012
	Mm237	1310	15	Chioggia	26.09.2012
	Mm240	1400	9	Chioggia	26.09.2012
	Mm247	1460	16	Chioggia	02.10.2012
	Mm266	1420	15	Chioggia	03.10.2012
	Mm274	1375	12	Chioggia	03.10.2012
	Mm275	1320	9	Chioggia	03.10.2012
	Mm278	1520	18	Chioggia	03.10.2012
	Mm279	1480	15	Chioggia	03.10.2012
	Mm280	1310	6	Chioggia	03.10.2012
	Mm288	1260	6	Mazara del Vallo	17.09.2012
*M. punctulatus*	Mp154	1255	27	Mazara del Vallo	12.2013
	Mp183	1250	34	Chioggia	29.08.2012
	Mp184	1290	35	Chioggia	29.08.2012
	Mp287	1250	14	Ancona	21.10.2008
	Mp3_14.7	1290	10	Chioggia	14.07.2011
	Mp4_14.7	1410	27	Chioggia	14.07.2011
	Mp7_14.7	1290	31	Chioggia	14.07.2011
	Mp1_6.9	1170	16	Chioggia	06.09.2011
	Mp4_6.9	1180	15	Chioggia	06.09.2011
	Mp6_6.9	1320	30	Chioggia	06.09.2011
	Mp7_6.9	1410	29	Chioggia	06.09.2011
	Mp9_6.9	1290	26	Chioggia	06.09.2011
	Mp1_21.9	1100	9	Chioggia	21.09.2011

**Table 3:** Summary statistics for 9 nuclear polymorphic microsatellite loci, for a total of 136 individuals of *Mustelus mustelus* and 117 of *M. punctulatus* from the northern Adriatic Sea and the southern Mediterranean Sea.

Locus	N_a_		H_o_		H_e_		HWE	
	Mm	Mp	Mm	Mp	Mm	Mp	Mm	Mp
MaD2X	5	3	0.654	0.479	0.675	0.530	0.703	0.504
McaB5	9	2	0.779	0.017	0.814	0.017	0.576	1.000
McaB35	13	3	0.852	0.385	0.831	0.315	0.325	0.025
Mh9	3	3	0.578	0.573	0.579	0.526	0.877	0.034
Mh25	6	4	0.184	0.615	0.218	0.659	0.012	0.588
Gg22*	12	—	0.743	—	0.788	—	0.416	—
MaFYP*	5	—	0.519	—	0.544	—	0.845	—
MaND5*	—	5	—	0.709	—	0.686	—	0.555
Mh29*	—	4	—	0.650	—	0.620	—	0.450

Reported are: locus name, total number of alleles (N_a_), observed (H_o_) and unbiased expected heterozygosity (H_e_) and HWE P-value for Hardy-Weinberg equilibrium. Mm = *M. mustelus*; Mp = *M. punctulatus*.

^*^Loci Gg22 and MaFYP amplified in *M. mustelus* only; loci MaND5 and Mh29 amplified in *M. punctulatus* only.

**Table 4:** Probability of detecting multiple matings (PrDM).

Mating scenario (paternal skew)	Litter size							
	*M. mustelus*				*M. punctulatus*			
	3	9	15	18	9	18	27	35
2 males (50:50)	46%	98%	100%	100%	75%	86%	87%	87%
2 males (66.7:33.3)	40%	95%	99%	99%	71%	84%	86%	87%
3 males (33.3:33.3:33.3)	60%	100%	100%	100%	90%	97%	98%	98%
3 males (57:28.5:14.5)	52%	98%	100%	100%	84%	95%	97%	98%

For both *Mustelus mustelus* and *M. punctulatus*, the table reports the PrDM obtained for distinct mating scenarios based on polymorphic loci of each species (see Table 3 for locus name) and litter sizes (3 to 18 embryos per litter in *M. mustelus*; 9 to 35 embryos per litter in *M. punctulatus*).

**Table 5:** Characteristics of the 16 polyandrous litters of *Mustelus mustelus* (N = 9 litters) and *M. punctulatus* (N = 7 litters).

Litter	TL (mm)	No. embryos	No. sires	Skew
Mm152	1630	10	2	8:2 (1.54E^−6^)
Mm159	1350	6	2	3:3 (5.76E^−8^)
Mm203	1550	16	2	3:12 (1.02E^−10^)
Mm229	1585	18	2	17:1 (4.14E^−10^)
Mm237	1310	15	2	14:1 (3.80E^−6^)
Mm240	1400	9	2	4:5 (3.54E^−5^)
Mm266	1420	15	2	4:10 (3.63E^−9^)
Mm274	1375	12	2	3:4 (3.61E^−3^)
Mm275	1320	9	3	4:3:2 (1.84E^−5^)
Mp154	1255	27	2	13:14 (1.9E^−11^)
Mp287	1250	14	2	5:9 (2.38E^−10^)
Mp1_6.9	1170	16	2	14:2 (4.09E^−10^)
Mp4_6.9	1180	15	2	6:9 (7.85E^−10^)
Mp6_6.9	1320	30	2	28:2 (1.42E^−9^)
Mp7_6.9	1410	29	2	14:15 (7.8E^−10^)
Mp9_6.9	1290	26	2	18:5 (NA)

For each litter the table reports maternal total length (TL), litter size (No. embryos), minimum number of sires suggested by GERUD (No. sires), most likely ratio of paternal contribution (Skew) and the relative probability (in parentheses). NA, no alternative.
