# Supplementary material for: Multiple paternity and hybridization in two smooth-hound sharks
Source: Sci Rep. 2015 Aug 10;5:12919. doi: 10.1038/srep12919 (PMC4530440; doi:10.1038/srep12919)
Supplement: Supplementary Information [file srep12919-s1.pdf]

Multiple paternity and hybridization in two smooth-hound sharks

Ilaria A. M. Marino[1], Emilio Riginella[1], Michele Gristina[2], Maria B. Rasotto[1], Lorenzo Zane[1*], Carlotta Mazzoldi[1]

[1] Department of Biology, University of Padova, Via U. Bassi 58/B, 35131 Padova, Italy

[2] IAMC-CNR, via Luigi Vaccara 61, 91026 Mazara del Vallo (TP), Italy

[*]Corresponding author, lorenzo.zane@unipd.it, phone: + 39 0498276220, fax: + 39 0498276209

Figure S1: Results of ITS2 assay for family Mp1_6.9. The figure presents the ITS2 amplicons, obtained following Marino et al. (2015), observed in the female (first row) and 16 embryos of the family Mp1_6.9. All the specimens, except two, showed the one band phenotype of *M. punctulatus* (Marino et al. 2015), with a fragment size of 161 bp highlighted in the first sample (notice the additional +1 bp peak due to the addition of one base during PCR, Clark 1988); two embryos (Mp1_6.9_E7 and Mp1_6.9_E14) showed a two band phenotype, with the 161 bp band of *M. punctulatus* and another band at 185 bp (and its additional PCR +1 base pair peak). Considering that the 185 bp band is typical of *M. mustelus* (Marino et al. 2015), this result support the hybrid nature of these two pups.

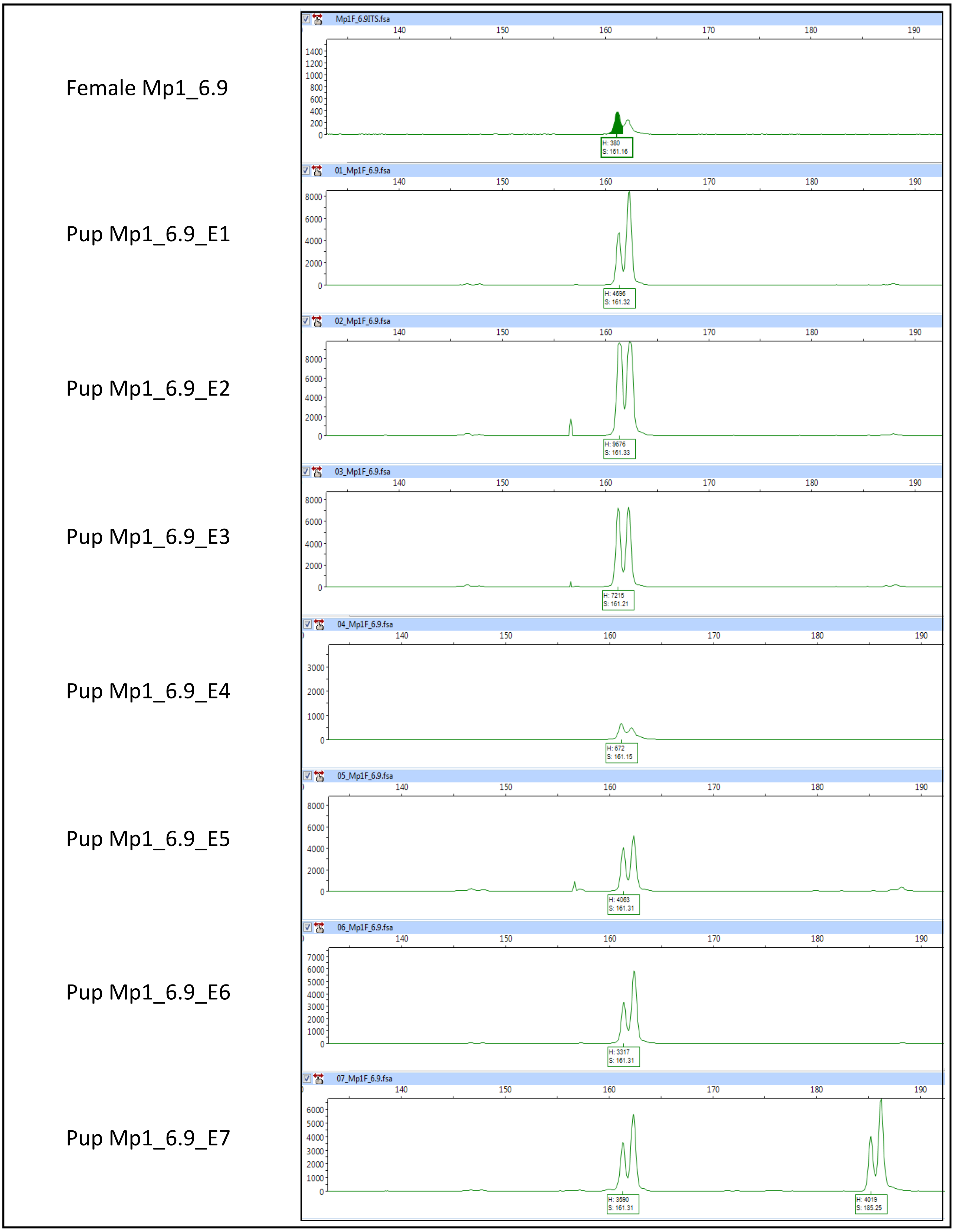

Figure S1 (continued): Results of ITS2 assay for family Mp1_6.9. This panel reports the profiles for pups Mp1_6.9_E8 to Mp1_6.9_E16 all, except one, showing the 161 bp band of *M. punctulatus*. Mp1_6.9_E14 shows the two band phenotype, with the 161 bp band of *M. punctulatus* and the 185 bp of *M. mustelus*.

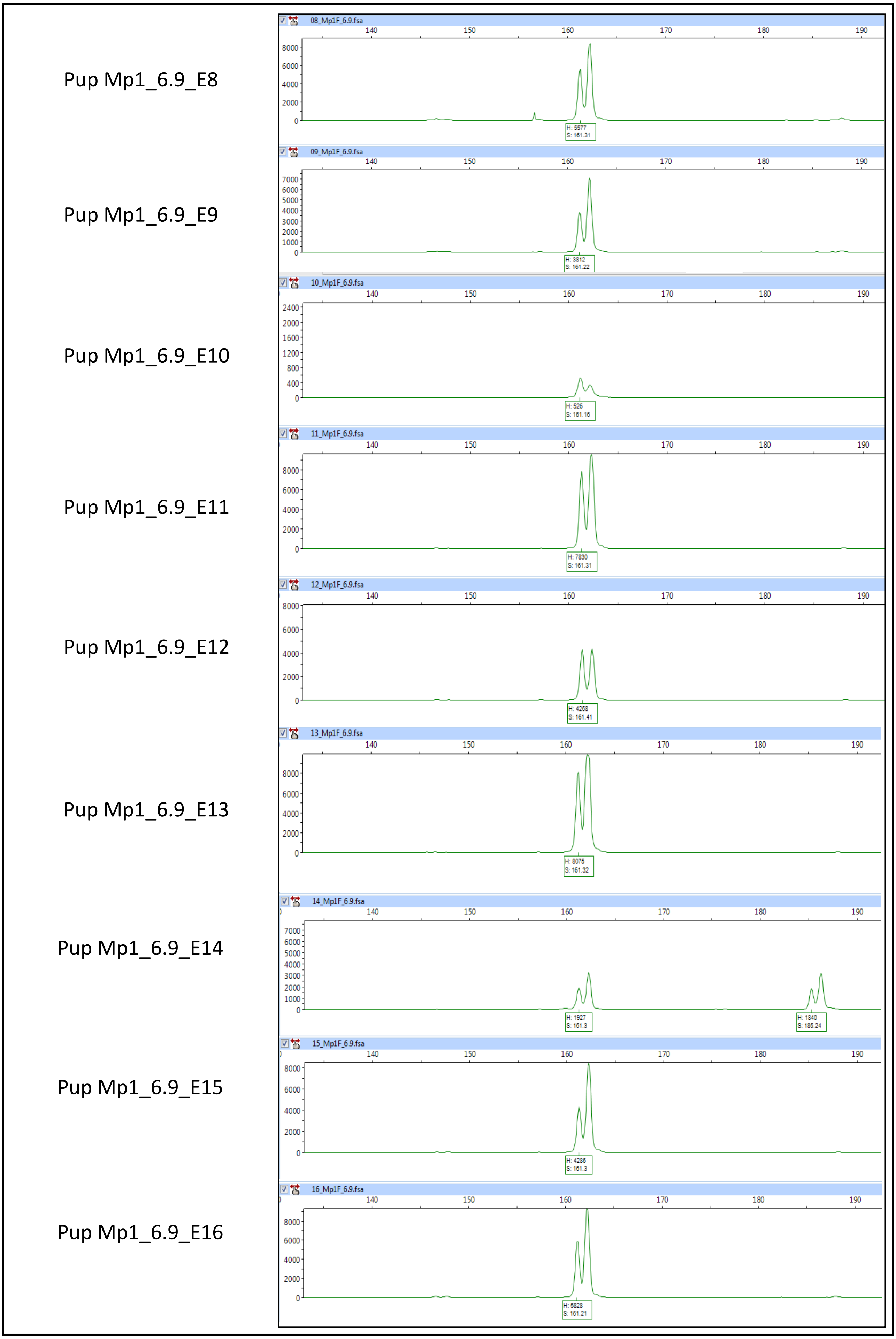

Figure S2: Admixture analysis of 270 individuals (family Mp1_6.9 with 16 pups, 136 adults of *Mustelus mustelus* and 117 adults of *M. punctulatus*) estimated from four loci (MaD2X, McaB35, Mh9, Mh25, successfully amplified and most polymorphic in both species) using the software Structure (Pritchard et al. 2000, Falush et al. 2003, Hubisz et al. 2009). Each individual is represented by a vertical line, which is partitioned into K coloured segments, the length of each colour being proportional to the estimated membership coefficient from cluster 1 (red, *M. mustelus*) and cluster 2 (green, *M. punctulatus*).

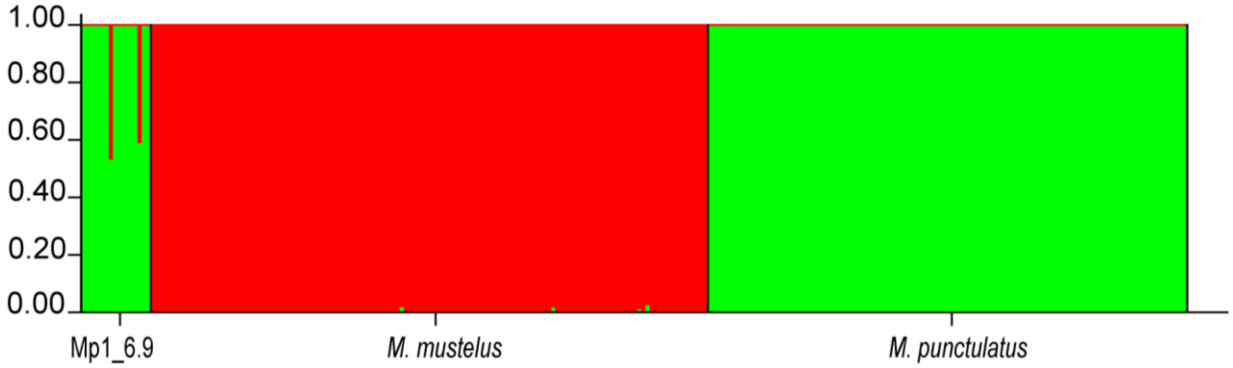

Table S1. Microsatellite loci used in this study. Reported are: locus name, primer sequences, species in which each primer pair works, and reference for the locus isolation. Six loci, highlighted in light blue (Gg4, Gg20, MaTJ5, Mca33, McaB26, Mh1), were used for molecular identification of all the 792 specimens analysed. Nine loci, highlighted in light orange (Gg22, MaD2X, MaFYP, MaND5, McaB5, McaB35, Mh9, Mh25, Mh29), were used to assess multiple paternity. Amplification conditions are described in Marino et al. (2015).

Locus	Primer sequence (5'-3')	Species	References
**Gg4**	F: CTGGAATACATGCCGAGCAC<br>R: CCCGAAAGGTCTTAGTTCGC	M. *mustelus* and *M. punctulatus*	Chabot and Nigenda, 2011
**Gg20**	F: GACCAAGGGTCATCCAGAC<br>R: TCAGCTTGGGCAATTCCAG	M. *mustelus* and *M. punctulatus*	Chabot and Nigenda, 2011
**MaTJ5**	F: TGCCTCTGTTATGCCCCTC<br>R: GGGGTCGAGAAGCATGTTG	M. *mustelus* and *M. punctulatus*	Boomer and Stow, 2010
**Mca33**	F: CATTTGAACCCCGACAGAAC<br>R: TCCAAGTAAGGATGAGTGACACC	M. *mustelus* and *M. punctulatus*	Giresi *et al.*, 2012
**McaB26**	F: ACTGTGGCACTGCATTCTGC<br>R: TGCATTTCAAAACCACTGGA	M. *mustelus* and *M. punctulatus*	Giresi *et al.*, 2012
**Mh1**	F: GGAGGAGGGAAGCCTATGG<br>R: TCTCTGGCTCCATTCAGGG	M. *mustelus* and *M. punctulatus*	Chabot, 2012
**MaD2X**	F: ACCTGGCCCAAGAACTCTC<br>R: ACTGGTGATGTGTGGACCC	M. *mustelus* and *M. punctulatus*	Boomer and Stow, 2010
**McaB5**	F: TAATCGACACGCAGTCATCG<br>R: AAGCTCCAATTCTCACTGTGC	M. *mustelus* and *M. punctulatus*	Giresi *et al.*, 2012
**McaB35**	F: AGTGCGTGCCAGTGTATGAG<br>R: GTTCTGCATGGGACGTGAC	M. *mustelus* and *M. punctulatus*	Giresi *et al.*, 2012
**Mh9**	F: CAACCATCTTTACTACACTG<br>R: GATGGACCTCACATTTAACAC	M. *mustelus* and *M. punctulatus*	Byrne and Avise, 2012
**Mh25**	F: TGCAATAACCGTTCTGCGTC<br>R: TCACACCCGCAGTTAGATCC	M. *mustelus* and *M. punctulatus*	Chabot, 2012
**Gg22**	F: TCCTGGGATGGCAACTTCG<br>R: AGGCCACCCAACTATCCTG	M. *mustelus*	Chabot and Nigenda, 2011
**MaFYP**	F: TGGTTGCCGATACAGCAGG<br>R: CAAGCGCATGCACACTCAC	M. *mustelus*	Boomer and Stow, 2010
**MaND5**	F: TGGGAGGCCAATGGATCAG<br>R: CGTTTCTGGGTGGTGCTTC	*M. punctulatus*	Boomer and Stow, 2010
**Mh29**	F: ATCAGCCCAGATTGTCCGC<br>R: AGACATTCCGCCTTCCAGC	*M. punctulatus*	Chabot, 2012

Table S2. Probability of detecting multiple matings. The table reports the probability of detecting multiple matings calculated with PrDM (Neff and Pitcher 2002) in *Mustelus mustelus* and *M. punctulatus* assuming 4 distinct mating scenarios and specific litter sizes. Reported are the species identification, female individual ID, litter size and mating scenarios, with red highlighting the females with multiple paternity.

Species	Mother	Litter size	PrDM (2 males 50:50)	PrDM (2 males skewed)	PrDM (3 males 33:33:33)	PrDM (3 males skewed)
Mustelus mustelus	Mm152	10	0.99	0.96	1.00	0.99
	Mm155	4	0.72	0.64	0.85	0.76
	Mm156	4	0.72	0.64	0.85	0.76
	Mm159	6	0.91	0.85	0.98	0.93
	Mm161	3	0.46	0.40	0.60	0.52
	Mm203	16	1.00	0.99	1.00	1.00
	Mm229	18	1.00	0.99	1.00	1.00
	Mm230	15	1.00	0.99	1.00	1.00
	Mm235	7	0.95	0.90	0.99	0.96
	Mm237	15	1.00	0.99	1.00	1.00
	Mm240	9	0.98	0.95	1.00	0.98
	Mm247	16	1.00	0.99	1.00	1.00
	Mm266	15	1.00	0.99	1.00	1.00
	Mm274	12	0.99	0.98	1.00	1.00
	Mm275	9	0.98	0.95	1.00	0.98
	Mm278	18	1.00	0.99	1.00	1.00
	Mm279	15	1.00	0.99	1.00	1.00
	Mm280	6	0.91	0.85	0.98	0.93
	Mm288	6	0.91	0.85	0.98	0.93
Mustelus punctulatus	Mp154	27	0.87	0.86	0.98	0.97
	Mp183	34	0.88	0.87	0.99	0.98
	Mp184	35	0.87	0.87	0.98	0.98
	Mp287	14	0.83	0.81	0.96	0.93
	Mp3_14.7	10	0.78	0.74	0.92	0.87
	Mp4_14.7	27	0.87	0.86	0.98	0.97
	Mp7_14.7	31	0.88	0.87	0.98	0.98
	Mp1_6.9	16	0.85	0.83	0.97	0.94
	Mp4_6.9	15	0.84	0.82	0.97	0.94
	Mp6_6.9	30	0.87	0.87	0.98	0.98
	Mp7_6.9	29	0.88	0.87	0.98	0.98
	Mp9_6.9	26	0.88	0.86	0.98	0.97
	Mp1_21.9	9	0.75	0.71	0.90	0.84

Supplementary Materials References

Boomer, J. J., and Stow, A. J. Rapid isolation of the first set of polymorphic microsatellite loci from the Australian gummy shark, *Mustelus antarcticus* and their utility across divergent shark taxa. *Conserv. Genet. Resour.* **2**, 393-395 (2010).

Byrne, R. J., and Avise, J. C. Genetic mating system of the brown smoothhound shark (*Mustelus henlei*), including a literature review of multiple paternity in other elasmobranch species. *Mar. Biol.* **159**, 749-756 (2012).

Chabot, C. L. Characterization of 11 microsatellite loci for the brown smooth-hound shark, *Mustelus henlei* (Triakidae), discovered with next-generation sequencing. *Conserv. Genet. Resour.* **4**, 23-25 (2012).

Chabot, C. L., and Nigenda, S. Characterization of 13 microsatellite loci for the tope shark, *Galeorhinus galeus*, discovered with next-generation sequencing and their utility for eastern Pacific smooth-hound sharks (*Mustelus*). *Conserv. Genet. Resour.* **3**, 553-555 (2011).

Clark, J. M. Novel non-templated nucleotide addition reactions catalyzed by procaryotic and eucaryotic DNA polymerases. *Nucl. Acids Res.* **16**, 9677-9686 (1988).

Falush, D., Stephens, M., & Pritchard, J. K. Inference of population structure using multilocus genotype data: linked loci and correlated allele frequencies. *Genetics* **164**, 1567-1587 (2003).

Giresi, M., Renshaw, M. A., Portnoy, D. S., Gold, J. R. Isolation and characterization of microsatellite markers for the dusky smoothhound shark, *Mustelus canis*. *Conserv. Genet. Resour.* **4**, 101-104 (2012).

Hubisz, M., Falush, D., Stephens, M., Pritchard, J. K. Inferring weak population structure with the assistance of sample group information. *Mol. Ecol. Resour.* **9**, 1322-1332 (2009).

Marino, I. A. M., et al. New molecular tools for the identification of two endangered smooth-hound sharks, Mustelus mustelus and Mustelus punctulatus. *J. Hered.* **106**, 123-130 (2015).

Neff, B.D., and Pitcher, T.E. Assessing the statistical power of genetic analyses to detect multiple mating in fishes. *J. Fish Biol.* **61**, 739-750 (2002).

Pritchard, J. K., Stephens, M., & Donnelly, P. Inference of population structure using multilocus genotype data. *Genetics* **155**, 945-959 (2000).
